# Evolution of Preclinical Models for Glioblastoma Modelling and Drug Screening

**DOI:** 10.1007/s11912-025-01672-4

**Published:** 2025-04-04

**Authors:** Grace Thomas, Ruman Rahman

**Affiliations:** Biodiscovery Institute, School of Medicine, University of Nottingham, Nottingham, NG7 2RD UK

**Keywords:** Glioblastoma, Preclinical model, Organoids, Microfluids, Glioblastoma-on-a-chip

## Abstract

**Purpose of Review:**

Isocitrate dehydrogenase wild-type glioblastoma is an extremely aggressive and fatal primary brain tumour, characterised by extensive heterogeneity and diffuse infiltration of brain parenchyma. Despite multimodal treatment and diverse research efforts to develop novel therapies, there has been limited success in improving patient outcomes. Constructing physiologically relevant preclinical models is essential to optimising drug screening processes and identifying more effective treatments.

**Recent Findings:**

Traditional in-vitro models have provided critical insights into glioblastoma pathophysiology; however, they are limited in their ability to recapitulate the complex tumour microenvironment and its interactions with surrounding cells. In-vivo models offer a more physiologically relevant context, but often do not fully represent human pathology, are expensive, and time-consuming. These limitations have contributed to the low translational success of therapies from trials to clinic. Organoid and glioblastoma-on-a-chip technology represent significant advances in glioblastoma modelling and enable the replication of key features of the human tumour microenvironment, including its structural, mechanical, and biochemical properties. Organoids provide a 3D system that captures cellular heterogeneity and tumour architecture, while microfluidic chips offer dynamic systems capable of mimicking vascularisation and nutrient exchange. Together, these technologies hold tremendous potential for high throughput drug screening and personalised, precision medicine.

**Summary:**

This review explores the evolution of preclinical models in glioblastoma modelling and drug screening, emphasising the transition from traditional systems to more advanced organoid and microfluidic platforms. Furthermore, it aims to evaluate the advantages and limitations of both traditional and next-generation models, investigating their combined potential to address current challenges by integrating complementary aspects of specific models and techniques.

## Introduction

### Pathophysiology

Isocitrate dehydrogenase (IDH) wild-type glioblastoma (GBM) is an aggressive brain tumour, arising from the malignant transformation of glial cells. GBM is the most common primary malignant brain tumour and remains incurable, with a median survival of 14.6 months from diagnosis [1]. Primary GBM initially develops from *de novo* processes and is typically more aggressive [2]. Pseudo-palisading necrosis and microvascular proliferation are key histological features that traditionally distinguish GBM from lower grade astrocytoma [3]. More recently, the 2021 WHO classification places a greater emphasis on molecular characteristics to enhance the diagnostic accuracy of GBM, including IDH wild-type status, Epidermal Growth Factor Receptor (EGFR) amplification and Telomerase Reverse Transcriptase (TERT) promotor mutations [3].

### Epidemiology /prognosis

The incidence of GBM increases with age, peaking between 75 and 84 years, with a median age of diagnosis of 64 years(2). GBM is slightly more common in men than women, with an incidence ratio 1.6:1 [4]. With a five-year survival rate less than 5%, GBM is amongst the lowest of all cancer types [4–5]. Even with standard multimodal treatment, approximately 70% of patients face disease progression within a year of diagnosis [4].

### Current Treatments and Associated Challenges

The current multimodal treatment regimen consists of maximal safe surgical resection, adjuvant with the Stupp protocol, which includes 6 weeks concurrent temozolomide (TMZ) chemotherapy and radiotherapy, followed by an additional 6 cycles of TMZ for 5 days every 28 days [6]. Although evidence suggests standard treatment does improve survival, there are a variety of distinct issues related to brain tumour treatment that impact efficacy [7].

**Fig. 1:**
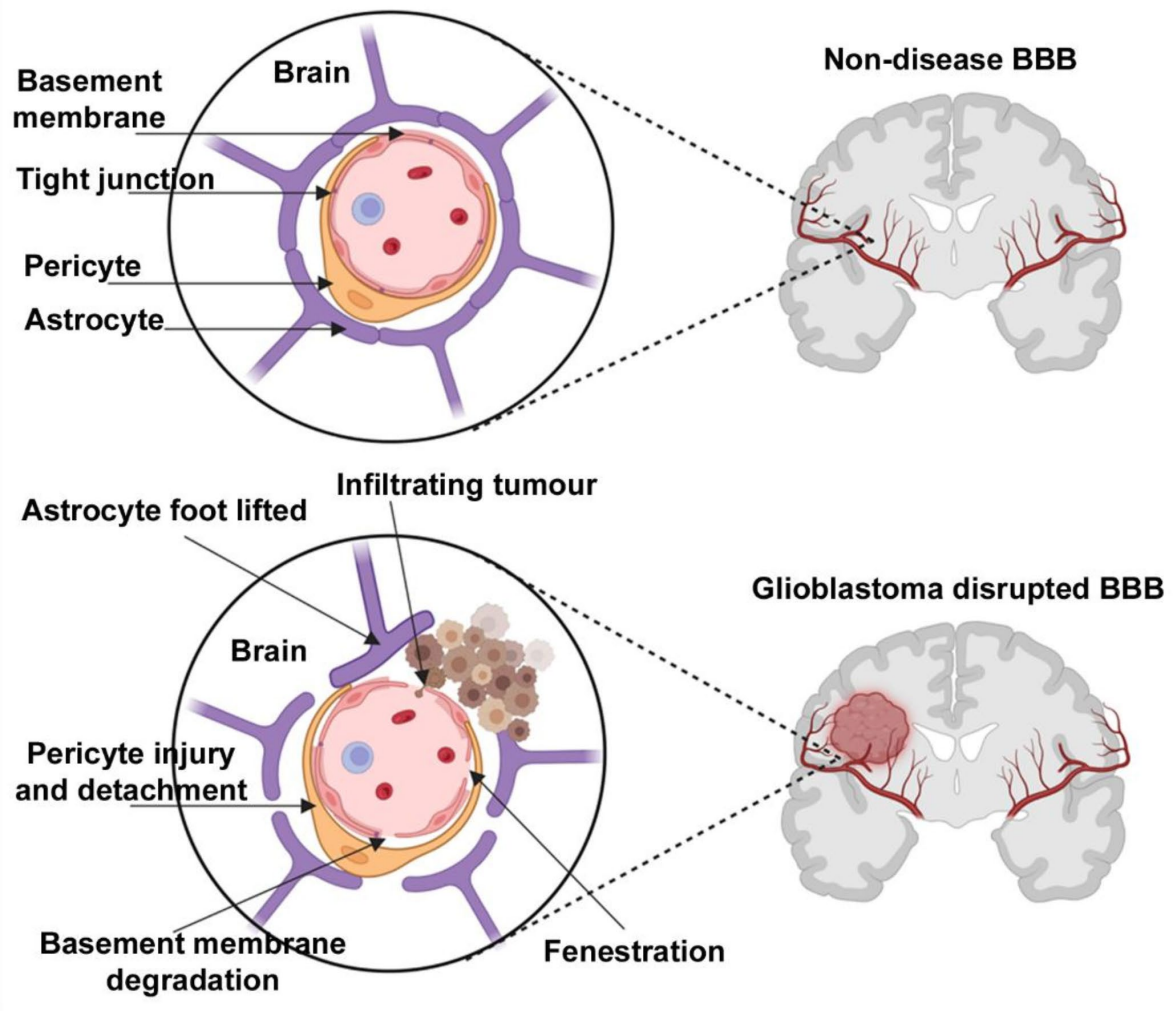
Healthy BBB versus a GBM disrupted BBB. GBM disrupted BBB characteristics of basement membrane degradation, infiltrating tumour cells, astrocyte foot lifted, fenestrations, loss of tight junctions, pericyte injury and detachment. Created with BioRender

The GBM tumour microenvironment (TME) is a dynamic and evolving ecosystem composed of both cellular and acellular elements that collectively promote tumour survival and growth [8]. Furthermore, the blood-brain barrier (BBB) is a semi-permeable membrane between the blood and brain interstitium, consisting of astrocytes and pericytes that make up tight junctions. Several fundamental structural proteins regulate tight junction permeability, such as claudin, a protein particularly relevant to GBM. The selective passageway of the BBB confers key immunological and homeostatic benefits by preventing the passage of neurotoxins and microorganisms into the central nervous system (CNS). However, the BBB also interferes with the transfer of drugs to the CNS, resulting in hindered efficacy of many therapeutic agents [6]. Paradoxically, the integrity of the BBB is significantly disrupted in many brain cancers, including GBM. Loss of claudin-3 and altered levels of claudin-1 and claudin-5 result in increased tight junction permeability, leading to extensive infiltration, an important hallmark observed in GBM [9]. Although interference of the BBB may appear beneficial for drug delivery, its disruption is not widespread, allowing survival of ‘shielded’ cancer cells [9]. Furthermore, loss of BBB integrity facilitates tumour invasion and progression by allowing infiltration of tumour cells, inflammatory mediators and tumour promoting factors [6, 9]. Consequently, the loss of BBB integrity contributes towards the highly invasive nature of GBM, complicating treatment and worsening prognosis (Fig. 1).

Glioma stem cells (GSCs) represent a minority population of cells within GBM and arise from the activation of oncogenic pathways in neuronal stem cells, glial progenitors, and glial cells [10]. GSCs have the ability to self-renew and differentiate into various cell types, giving rise to the heterogeneity observed in GBM [6]. GSCs are highly resistant to standard therapies due to several mechanisms, including their quiescent state which makes them less susceptible to traditional chemotherapeutic agents that target rapidly dividing cells [6, 10]. Additionally, GSCs overexpress drug efflux transporters, such as ATP-binding cassette transporter channels, that actively pump out chemotherapeutics, reducing their intracellular concentration and efficacy [6, 10–11]. Through the release of immunosuppressive and pro-proliferative cytokines in addition to other signalling factors, GSCs promote abnormal neovascularisation and immune evasion, therefore acting as key mediators in the development of the immunosuppressive, hypoxic niche in GBM [11–12].

The high recurrence rate in GBM can furthermore be largely attributed to inter-, and intra- tumoral heterogeneity. This refers to the molecular and genetic differences between and within tumours, respectively. Key molecular characteristics of GBM include TERT promoter mutations (found in ~ 80% of patients), and EGFR amplification (~ 50%) [13]. Additionally, mutations in TP53, PTEN and CDKN2A, and alterations in oncogenic pathways such as P21-RAS, and PI3k amongst others are commonly observed(13). The gain of chromosome 7 and loss of chromosomes 10 and 13 are common in GBM and mainly caused by neoplastic cells [14]. Evidence suggests the simultaneous gain of chromosome 7 and loss of chromosome 10 is associated with EGFR amplification and the PTEN deletion mutation, emphasising the relationship between chromosomal alterations and oncogenic drivers in GBM progression [14]. Treatment techniques such as combination, personalised and targeted therapies, can be employed in an effort to overcome heterogeneity. Combination therapy integrates multiple treatment agents and modalities, aiming to target different tumour mechanisms simultaneously, thereby reducing the likelihood of resistant cell populations. The use of personalised medicine considers a patient’s own molecular and genetic profile, enabling a tailored regimen [6]. Lastly, targeted therapies are used to directly target specific genetic alterations within the tumour whilst sparing healthy tissue. Despite these strategies, heterogeneity can still enable some cancer cells to evade therapy, driving tumour recurrence.

### Emerging Therapies

In light of the poor prognosis associated with GBM, there is a desperate need for research into innovative treatment options. Emerging approaches primarily involve immunotherapies and nanotherapies.

#### Immunotherapy

Immunotherapy stimulates the immune system to attack cancer cells, often inducing the abscopal effect. This is a phenomenon where localised treatment triggers a systemic immune response against cancer cells in sites that were not directly treated. Initially, immunotherapy had not been considered a feasible treatment for GBM as the brain was regarded an immunological sanctuary, partly due to the BBB serving as an immunological barrier [9]. It is now recognised that the brain is under constant immune surveillance and communicates with the peripheral immune system via meningeal lymphatic vessels that drain into the deep cervical lymph nodes(9). Check point inhibitors (CPIs), vaccines and chimeric antigen receptor T (CAR-T) cell therapy are novel immunotherapies that show promise for future treatment.

Immune checkpoints are cell surface receptors that control and restrict immune activity to avert autoimmunity. Cancer cells can evade immune destruction by enhancing expression of immune checkpoints, including programmed cell death protein-1 (PD-1) and cytotoxic T-lymphocyte-associated protein 4 (CTLA-4) [15]. CPI treatment utilises monoclonal antibodies to modulate immune response and have proven highly effective in the treatment of many cancers. Despite the preclinical promise shown, clinical trials for CPI treatment in GBM have thus far demonstrated limited efficacy; more research is required to determine their potential therapeutic role in GBM [15–17].

Vaccine based therapeutics aim to prime the immune system to better recognise tumour associated antigens (TAA), that are present on all cells but are overexpressed in cancer cells, and tumour specific antigens (TSA) [10]. This enables the immune system, specifically cytotoxic T-lymphocytes (CTLs), to mount a targeted response that leads to the destruction of cancer cells by inducing apoptosis. GBM vaccine therapy is currently being investigated as a potential therapeutic, with three main approaches under exploration:

i. Peptide based vaccines: utilise synthetic or naturally derived peptides from TAA or TSA GBM antigens to activate the immune system [10].
ii. Dendritic cell (DC) vaccines: patient isolated DCs are exposed to TSA ex vivo, then reintroduced into the patient to prompt an immune response [18].
iii. Viral vector vaccines: employ natural or genetically modified viruses to deliver tumour antigens, attack cancer cells directly and modify the TME, thereby enhancing immune activity [18].

Clinical trials for vaccine therapy are also unfortunately generating efficacy results lower than expected [19]. CAR-T therapy is an advanced form of personalised immunotherapy, involving the isolation of T lymphocytes from a sample of the patients’ blood and genetically modifying the cells to express chimeric antigen receptors (CARs) [18]. CAR-T cells therefore possess both an extracellular TSA recognition domain (the CAR) and an intracellular T-cell activation domain [10]. These cells are then intravenously, intratumorally or intracranial injected back into the patient where they are able to specifically identify and bind to cancer cells, triggering direct tumour cytotoxicity and the release of cytokines that disrupt the TME [10, 18]. CAR-T therapy has been incredibly successful in the treatment of certain haematological cancers, hence there is currently extensive research in progress aimed at translating this success into effective therapies for solid cancers such as GBM. Several clinical trials are currently investigating GBM CAR-T cell target receptors, including human epidermal growth factor receptor 2 (HER2), EGFRvIII, interlukin-13 receptor alpha 2 (IL-13Rα2) and cluster of differentiation 70 (CD70) [10].

#### Nanotherapy

Nanoparticles (NPs) (typically < 200 nm), such as polymeric NPs, inorganic NPs and lipid-based NPs, can be leveraged as drug delivery systems and diagnostic tools. The specific characteristics of NPs, including size and surface charge, can influence its behaviour [10]. For instance, smaller and more positively charged NPs are readily able to cross the BBB and glioma cell membranes, facilitating their accumulation within the tumour [20]. However, these properties also give rise to toxicity due to their ability to enter the cytoplasm of normal cells, damage mitochondria, DNA and RNA, as well as generate toxic reactive oxygen species [10, 20]. By incorporating tumour specific ligands on their surfaces, NPs can achieve greater therapeutic precision, minimising systemic toxicity [10]. Hence, the careful incorporation of NPs into established and novel therapies, could address many current treatment limitations.

A major limitation of chemotherapy in GBM is its suboptimal BBB penetration, resulting in low drug concentrations at the tumour site and high systemic toxicity [21]. NPs can in principle improve drug delivery by encapsulating chemotherapeutic agents and thus minimising off-target effects. Additionally, NPs can enable the sustained release of therapeutics, therefore improving efficacy by maintaining therapeutic concentrations at the specific tumour site [20]. Radiotherapy can also benefit from NP incorporation as metal NPs can serve as radiosensitisers, enhancing tumour sensitivity to radiation by absorbing and focusing large amounts of radiation energy on the tumour site [21].

Although relatively novel, especially in the context of GBM treatment, NPs can be incorporated into immunotherapies to improve outcomes. Evidence suggests antigen-capturing NPs used in conjunction with CPIs can enhance their efficacy by improving antigen presentation. Similarly, preclinical trials suggest vaccine-based therapies can improve from the incorporation of NPs as they are able to protect against vaccine degradation, thus increasing antigen presentation and therapeutic outcomes. In CAR-T therapy, NPs have demonstrated the potential to operate as the antigen presenting portion and have shown to reduce costs, improve expansion and act as live T-cell trackers. Furthermore, NPs can encapsulate the T-cells and be modified to manage their release. Further research is required to confirm if NP therapy can improve immunotherapy outcomes enough to justify its widespread clinical application in GBM treatment [21].

Gene therapy is an innovative approach that shows promise as a treatment for GBM, leveraging advanced gene editing techniques such as CRISPR-Cas9, small interfering RNA (siRNA) and micro-RNA (miRNA). The incorporation of NPs may improve the safety, efficacy and specificity of gene targeting. Notably, CRISPR-Cas9 is a nuclease system that has recently gained attention as a favourable genome editing technique. Cas9 cleaves DNA at targeted sites, steered by an engineered single guide RNA (sgRNA), forming gene insertions or deletions. Although traditional delivery systems, such as viral vectors, exhibit high transduction efficiency, these pose safety concerns; therefore, clinical translation has been poor. As an alternative, liposome-templated NPs can be utilised to deliver Cas9 and sgRNA safely and efficiently to the target tumour site. Many preclinical trials are underway evaluating the role of gene therapy with NPs in overcoming the challenges of current treatment and improving outcomes in GBM patients [21].

### Preclinical Models

Preclinical models are crucial to understand the GBM pathology and to investigate numerous factors as potential therapeutics before clinical phase testing, including safety, side effects, dosage, and efficacy. In an ideal experimental setting, GBM preclinical models should fulfil the following requirements: (i) genetic profile and intratumoural heterogeneity mirrors human GBM; (ii) simulate the TME and its relationship with the human brain; (iii) is reproducible and stable over time [22].

Traditional preclinical models used for GBM drug screening include both in-vitro / ex-vivo and in-vivo models, each with unique advantages and limitations. The various models discussed below will be further explored in the section ‘Traditional preclinical models’.

In-vitro models consist primarily of 2D cell cultures derived from either established or patient derived cell lines [23]. A variety of different cell lines can be used and manipulated to study specific aspects of GBM and assess future therapeutics. Although 2D cell cultures are highly cost-effective in comparison to other models, they show limitations in accurately replicating the TME, hence reducing the reliability of these studies. 3D culture systems, including scaffolds, spheroids and more recently, cerebral organoids (COs), better mimic the TME by preserving cellular architecture and intercellular interactions, therefore offering a more clinically accurate insight. Additionally, ex-vivo models such as tumour tissue explants grown in a collagen-coated Petri dish conserve the structure and microenvironment of the tumour. However, these models are difficult to preserve and show poor reproducibility [24].

In-vivo models allow for the assessment of therapeutics in a living organism. The majority of preclinical in-vivo GBM studies utilise murine models that are adapted using various approaches. There are three current preclinical murine model categories: xenografts, genetically engineered mouse models (GEMMs) and syngeneic murine models [25]. Whilst murine models provide a greater understanding of the molecular and cellular oncogenic pathways and systemic drug effects, their use also raises significant ethical concerns. Traditional model systems have immensely contributed to the current knowledge and treatment of GBM; however, their limitations restrict further research and clinical applications.

Emerging next-generation models represent significant advancements in GBM preclinical modelling by addressing the limitations of conventional models. Recent developments, including COs and organ-on-a-chip (OoC) models with microfluidics and bioprinting offer more sophisticated representations of the GBM TME and architecture, allowing for a more accurate understanding of GBM and potential therapeutic targets.

This review provides a comprehensive overview of the advancements in preclinical models for GBM drug screening, with a specific emphasis on the next generation of models. Additionally, the review addresses the challenges associated with these advanced platforms, including issues of scalability, standardisation, and costs. By critically evaluating these features, it aims to highlight both the potential and the limitations of the next generation of preclinical GBM models, underscoring their significance in transforming GBM research and improving patient outcomes.

## Traditional Preclinical Models

### In-vitro Models

**Fig. 2:**
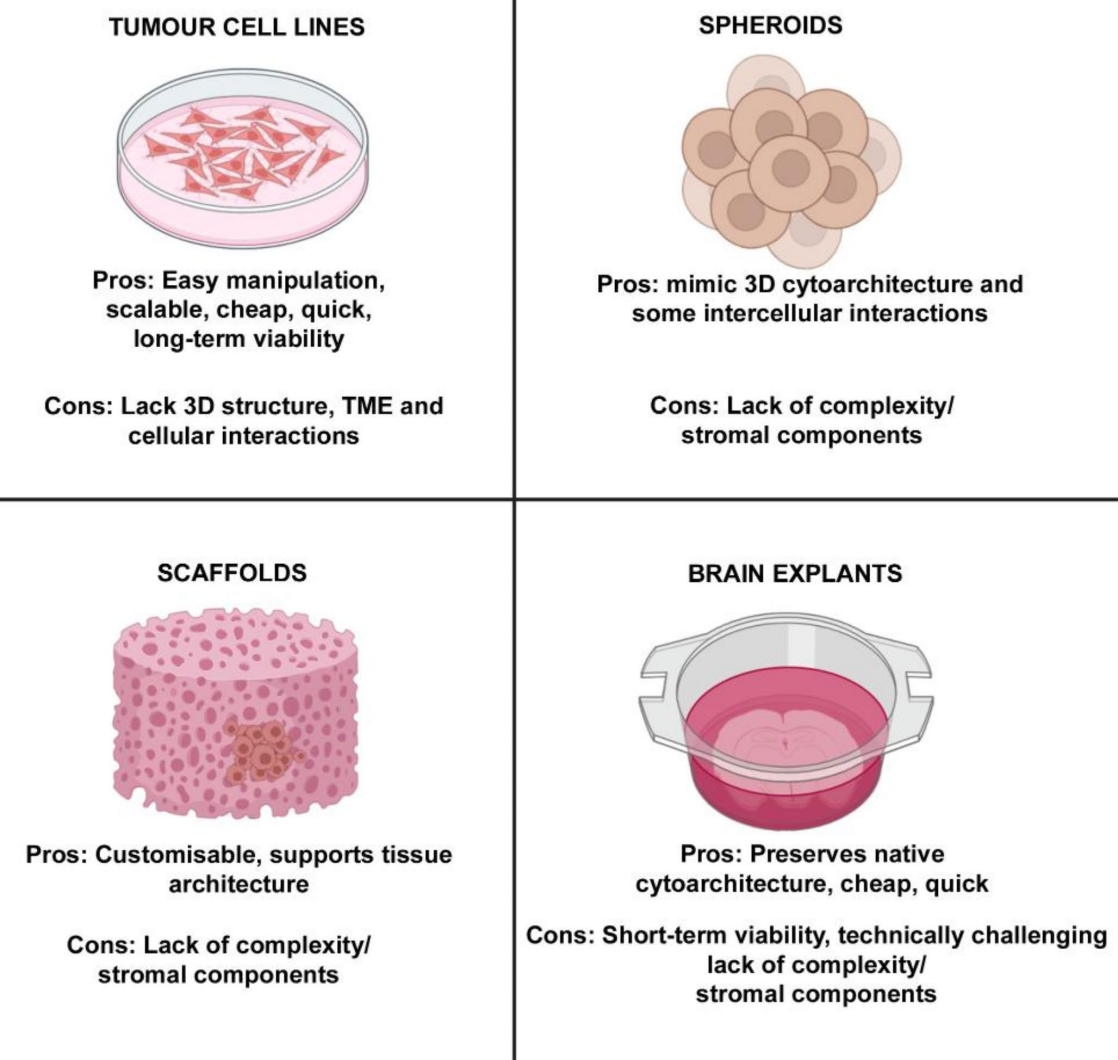
Overview of traditional in-vitro models for GBM research. Created with BioRender

An overview of advantages and disadvantages of well-characterised in-vitro models for GBM research are highlighted in Fig. 2.

#### Tumour Cell Lines

Immortalised cell lines are widely used in cancer research and can be generated by manipulating both normal and tumour cells in-vitro. A variety of cell lines have been established, as summarised in Table 1, the first of which was developed through the administration of nitrosourea to pregnant rats, inducing brain tumours in their offspring(26). Among these include the 9 L, C6 and F98 gliomas, which are some of the most commonly utilised rat cell lines in GBM/high-grade glioma research [26]. In contrast, the GL261 cell line originates from C57BL/6 mice injected intracranially with 3-methylcholantrene and is primarily employed in GBM immune research due to its murine origin [23]. Human-derived cell lines such as U87 and U251, were introduced in the late 1960s and have proven invaluable in the investigation of basic GBM biology, drug discovery and drug screening [27]. However, studies suggest many established cell lines used in research now show evidence of cross-contamination, reducing the reliability of these models [28]. Furthermore, it is important to recognise the phenotypic and genotypic drift that develops overtime with established cell lines, making it harder to predict therapeutic outcomes when drug screening.

**Table 1:** Summary of historically established GBM cell lines [23, 26]

Cell line	Strain of origin	Mode of origin	Molecular markers	Uses in evaluating		Advantages		Disadvantages	
C6	Wistar rats	Exposed astrocytes to N-methyl nitrosourea	Wild type Tp53, GFAP+, Increased expression of: PDGF-ß, EGFR, IGF-1, Erb3/Her3 precursor proteins, TGFα precursor, Rb gene, Ras Reduced expression of: FGF-9, FGF-10	Antiangiogenic~, Chemo~, Radio~, Oncolytic viral~, Gene~, Photodynamic~, Proteosome inhibitor~, (~ therapy)		Close molecular profile to human GBM		Triggers allogenic immune response, Well demarcated boarders, IDH-1 and IDH-2 mutations absent	
9 L	Fischer rats	i.v. administration of N-methyl nitrosourea	Mutant Tp53 gene Increased expression of: TGFα, EGFR Reduced expression of: FGF-2, FGF-9, FGFR-1, and PDGF-β	PET and MRI studies investigating tumour hypoxia and metabolism, Chemotherapeutic resistance, Drug transportation, Antiangiogenic ~, Chemo~, Radio~, Boron neutron capture~, Immunotoxin~, Oncolytic viral~, Immuno ~, (~ therapy)		Can also model brainstem tumours		High immunogenicity	
F98	Fischer rats	i.v. administration of N-ethyl nitrosourea	GFAP+, Vimentin + Increased expression of: PDGF-β, Rb, Ras, EGFR, cyclin D1, and cyclin D2	EGFR molecular targetting, Diffusion tensor imaging, Radio-iodine ~, Iodine-enhanced, Synchrotron sterotactic radio~, Chemo~, Suicide gene ~, Immuno ~, (~ therapy)		Weak immunogenicity, High invasive potential, High mitotic index, Necrotic core, Neovascular proliferation			
RG2	Fischer rats	i.v. administration of N-ethyl nitrosourea	Wild type Tp53, Increased expression of: PDGF-β, IGF-1, Erb3/Her3 precursor proteins, Ras, and D2 Loss of expression of: p16/Cdkn2a/Ink4 gene locus	Vascular permeability, BBB permeability, Chemo ~, Radionuclide ~, Antiangiogenic ~, Gene ~, Oncolytic viral ~, (~ therapy)		Weak immunogenicity, High invasive potential,			
GL261	C57BL/6 mice	Intracranial injection of 3-methylcholantrene	Wild type IDH1, GFAP-, Vimentin + Increased expression of: p14, p16, PTEN, K-ras, EGFR Reduced expression of: H-ras	Immunotherapy		Weak Immunogenicity, Pseudo-palisading necrosis, Perivascular proliferation, Nuclear pleomorphism		Distinct growth patterns High mutational load	
CT-2 A	C57BL/6 mice	Induction with methylcholanthrene	Wild-type Tp53 and IDH1, GFAP + Reduced expression of: PTEN	Immunotherapy		High Invasive potential, High mitotic index, Increased cell density, Nuclear polymorphisms, Haemorrhagic areas, Pseudo-palisading necrosis, High angiogenesis, Microvascular invasion,		Distinct tumour borders	
SB28	C57BL/6 mice	Transfection of the Sleeping Beauty transposon-flanked proto-oncogene		Immunotherapy		Low mutational load, Weak immunogenicity,		Homogenous population of cells, Limited use and not well characterised	
SMA-560	VM/Dk mice	Spontaneous	GFAP + Increased expression of: TGF-ß	Immunotherapy		Spontaneous development is more representative, Focal necrosis		Homogenous population of cells, Limited availability has led to gaps in literature, Not well characterised	
U87	Human		Wild-type Tp53 and IDH1, GFAP-, Vimentin+, Increased expression of: p14, p16, PTEN, kRAS Reduced expression of: p53, EGFR	Chemotherapy		High colony formation and migration potential		Weak vascularisation, Distinct tumour borders	
U251	Human		GFAP+, Vimentin + Increased expression of: p14, p16, PTEN, p53, kRAS, EGFR	Chemotherapy		Diffuse infiltration pattern, Winding vascular proliferation pattern, Nuclear pleomorphism, Haemorrhage, oedema		Does not exhibit invasive pattern through white matter tracts	

Patient-derived cell lines better reflect the genetic and molecular diversity of the original tumour. This enhanced representation of tumour heterogeneity facilitates the development of personalised therapeutic approaches. Unfortunately, this approach is costly and time-consuming, limiting its practicality as a model in wider research.

Each cell line derivative has distinct advantages and limitations and therefore should be selected cautiously depending on the nature of the study and given research question. Ultimately, 2D models provide an easily manipulatable system; however, they fail to accurately represent the TME and cellular interactions. As a result, there has been extensive progress and increasing adaptation of 3D in-vitro models.

#### Spheroid cancer Models

Spheroid cancer models (SCMs) mimic the behaviour of solid tumours and are one of the most utilised 3D cultures for GBM research [29]. SCMs are defined by their 3D sphere-like shape, presence of almost exclusively cancer cells, and their ability to proliferate in suspension. There are four recognised categories of SCMs used in cancer studies, each derived from different cancer cell sources and preparation techniques [30].

Multicellular tumour spheroids (MCTS) were the first SCM employed for glioma research in 1989. MCTS are developed by cultivating cancer cells in non-adherent conditions, such as liquid overlay or spinner flasks, to promote spontaneous self-aggregation and growth [23].

Currently, tumourspheres are the most widely used SCMs in glioma research largely due to their simplicity and ability to develop diffusely infiltrating tumours. In the early 2000’s tumourspheres were successfully developed from putative GSCs cultured with neurotrophic growth factors. Initially, tumourspheres were generated from a single cell suspension proliferating by clonal expansion; tissue derived cancer cells, circulating cancer cells and established cell lines are also now commonly utilised [23, 30].

The third SCM is organotypic multicellular spheroids (OMS), which are obtained from ex-vivo tumour fragments cultured without dissociation [30]. OMS accurately represent the TME and original matrix composition for up to 70 days of culture [31]. Furthermore, OMS can be frozen and still maintain their histological characteristics with only slight phenotypic and genotypic changes post thawing [30]. A major limitation of these models is the lack of GBM tissue available, restricting its wider use in GBM research.

Tissue-derived tumour spheres are the final SCM. In contrast to OMS, they are derived from partially dissociated cancer tissue [30]. However, this model has not yet been used in GBM research.

Spheroids overcome some limitations of 2D in-vitro models as they more accurately represent the 3D architecture, cell-cell and cell-ECM interactions exhibited in solid tumours [32]. Although spheroids mimic certain aspects of the TME, they often lack the full complexity of in-vivo systems including interactions with immune cells, endothelial cells, and stromal components [23].

#### Scaffolds

Scaffolds are 3D frameworks introduced to cell cultures to provide support and structure. Scaffold models can be synthetic or natural, with varying properties such as stiffness, porosity, interconnectivity, and structural integrity, collectively influencing cell behaviour [27, 31]. Hence, careful selection of scaffold materials and consideration of their properties is essential to devise a representative model. Studies suggest that 3D collagen scaffolds effectively simulate the high proportion of fibrillary collagens exhibited in the GBM ECM(31). 3D collagen scaffolds also demonstrate increased stemness and greater resistance to chemotherapy compared to 2D models, thereby more accurately replicating GBM morphology [33].

Traditionally, scaffolds were generated using techniques such as electrospinning, hydrogel formation and temperature-induced phase separation [27, 31]. However, these methods often lack precise control over scaffold architecture, mechanical properties, and reproducibility, resulting in the development of more advanced fabrication technologies. Solid free-form technologies represent significant progression in scaffold fabrication and include technologies such as 3D and, most recently, 4D bioprinting [23].

3D bioprinting involves the use of bio-inks, which are specially formulated materials that can contain living cells, growth factors and ECM components, guided by a computer aided design to layer-by-layer deposit these materials with high precision. There are two main types of bio-inks: scaffold-base bio-inks and scaffold-free bio-inks. Scaffold-base bio-inks incorporate soft biomaterials, such as hydrogels, whereas scaffold-free bio-inks are composed primarily of living cells [31, 34]. 3D bioprinting has transformed our knowledge of GBM by enabling researchers to create highly specific and manipulatable models, allowing for extensive research into tumour progression and more effective drug screening [35].

4D bioprinting is a novel technique, developed from improvements in 3D bioprinting that introduce the dimension of time. This is achieved by using dynamic bio-inks and materials capable of responding to external stimuli. In the context of GBM research, 4D bioprinting holds immense potential for accurately mimicking the dynamic nature of the TME, simulating the morphological adaptations of tumours to changing conditions. By enabling such precise simulations, 4D bioprinting provides a deeper insight into tumour behaviour and response to treatment [36, 37].

#### Brain Explants

Pre-mortem brain samples, obtained through surgical resections or biopsies, are utilised not only in diagnosis, but also in advancing research and analysis of GBM. Unlike post-mortem tissues, pre-mortem samples typically have minimal degradation and so are a promising model for GBM studies. However, pre-mortem samples fail to fully capture the spatial and temporal heterogeneity of GBM or provide insight into end-stage disease, therefore limiting their application [38].

Post-mortem brain samples have gained increasing approval as models in GBM research, particularly as they are able to represent end-stage disease. As such, these samples have contributed immensely to the current knowledge of the molecular and cellular pathways underlying GBM, including the mutational changes that drive recurrence. The impact of post-mortem interval (PMI) on tissue quality is largely debated in the literature. Although the true extent of PMI is still unknown, it is widely accepted that high quality samples can be acquired; however, this data cannot directly be extrapolated to GBM samples. Further limitations of post-mortem samples include high costs and lack of donors [38].

The development of organotypic brain slice cultures (OBSCs) has emerged as a powerful tool in GBM research. This technique involves resecting a healthy slice of brain tissue, typically from mouse or rat brains, although healthy human tissue from the tumour periphery can also be utilised and often better reflects the patient specific TME. These slices are cultured in media (typically neurobasal medium) for at least twenty-four hours, after which isolated cells or spheroids can be incorporated. OBSCs are particularly useful because they preserve the native vasculature and immune components, which are often absent in alternative traditional in-vitro models. This capability facilitates the study of GBM migration, tumour-immune interactions, and the effects of therapeutics on these processes. Furthermore, organotypic cultures offer researchers greater flexibility, allowing them to tailor the model to meet specific research goals. This is achieved through the application of small molecule inhibitors or by using GEMMs as tissue sources, enabling precise modifications to both tumour cells and the surrounding microenvironment. OBSCs are cheaper and less time-consuming than in-vivo models but can lack specific components of the TME if only tumour cells are transplanted [39, 40].

### In-Vivo Models

Animal models in GBM research have progressed immensely since their first use in the 1940s [23]. Murine models are the most widely utilised animal model due to their accessibility and lower costs. Additional animal models include, but are not limited to, canine, non-human primates, and pig models; however, their use is largely restricted primarily because of ethical concerns [23]. Hence, this section specifically focuses on the variety of murine models used in research.

**Fig. 3:**
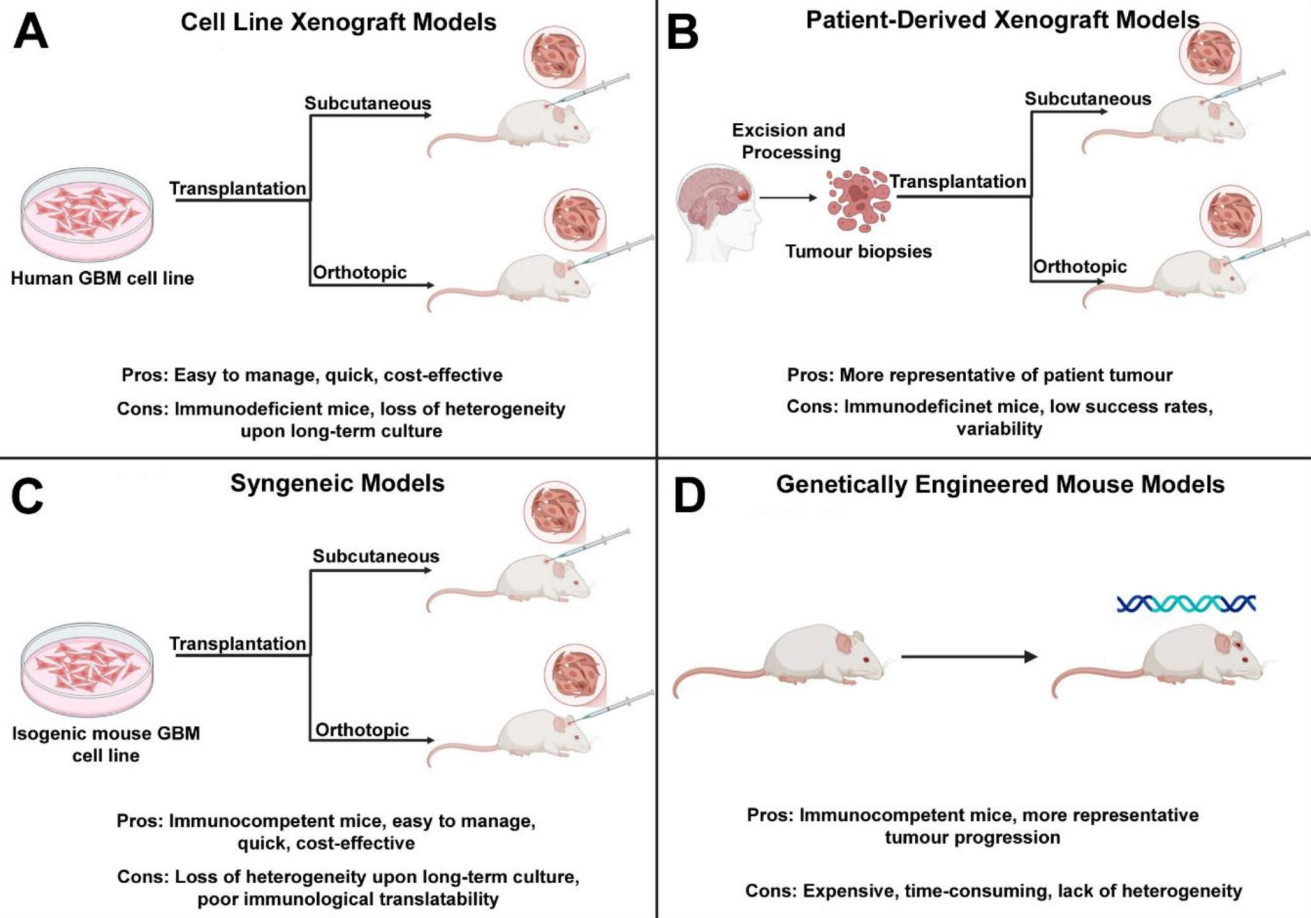
Overview of traditional in-vivo mouse models for GBM research. Created with BioRender

It is essential to recognise the anatomical and functional differences in mouse brains compared to humans. The mouse brain lacks the gyration and cortical properties of the human brain, limiting its translational function [23]. When using mice as a preclinical model for drug screening, it is vital to consider these differences and adjust trial designs accordingly to ensure their safe application in clinical trials. Regardless, animal models have dramatically contributed to the current understanding of GBM pathology and treatment, substantiated by their continued use and development in research (Fig. 3).

#### Xenograft Models

Xenograft models are generated by implanting human GBM tumour cells into immunodeficient mice either subcutaneously or orthotopically. Xenograft models allow investigations into human specific tumour biology and treatment response in an in-vivo context and therefore have contributed substantially to research. Additionally, their flexibility, speed of tumour development and cost-effectiveness, offer significant advantages over alternative in-vivo models. Nevertheless, a major drawback of xenograft models is their restriction to immunodeficient mice. This limits their use in evaluating immunotherapies, a key therapeutic currently under research for GBM. Xenograft models can be categorised into GBM established cell line xenografts and primary patient derived xenografts (PDXs) (Fig. 3A-B).

As previously stated, cell lines are convenient to manage and can be preserved for long periods of time, making them ideal models to research tumour signalling pathways, novel therapeutics, and mechanisms of resistance. Indeed, many of the advantages and disadvantages of in-vitro cell lines are reflected in cell line xenograft models, with the obvious added aspects of in-vivo modelling (Fig. 3A). The U87 and U251 cell lines have commonly been used in mice xenograft GBM models, but increasingly, primary patient-derived cell lines are considered the most clinically-relevant PDX cellular source [41].

PDXs involve the isolation of tumour tissue from a patient, processing into pieces or a single cell suspension and immediate injection into an immunosuppressed mouse [41]. In comparison to cell line xenografts, PDXs better retain the molecular and phenotypic characteristics of the original tumour as they are not subjected to prolonged cell culture. It is for this reason that PDXs are considered one of the most beneficial GBM models to date [25]. However, it is still important to acknowledge the limitations of PDXs. Even though there are a range of techniques available, success rates in establishing PDXs can vary. Variability between models can limit standardisation and reproducibility of results [41] (Fig. 3B).

There is major debate as to whether orthotopic or subcutaneous xenografts are superior. Subcutaneous xenografts involve the injection of processed tumour cells directly into the flank of immunodeficient mice and are thereby deemed less challenging and easier to monitor than orthotopic xenografts. However, the key advantage of orthotopic xenografts, whereby the cells are implanted specifically into the affected organ, is that they more accurately model the TME.

#### Syngeneic Models

Syngeneic mouse models consist of the transplantation of isogenic GBM tumour cells derived from the same genetic background as the given mouse strain (Fig. 3C). There are three common methods of developing syngeneic tumour lines: spontaneously occurring murine tumours, mutagenic chemicals and transposons [41, 42]. One of the most frequently used syngeneic mice models is the GL261 model, which was generated in 1970 via chemical induction. This model has demonstrated validity due to its close resemblance to human GBM, mimicking histology with high levels of accuracy [43]. Other regularly used syngeneic models include SB28, CT-2 A and SMA-560; each possess unique advantages and limitations, as displayed in Table 1, and so should be carefully selected to align with research goals.

Syngeneic models utilise cell lines and therefore are susceptible to the stresses that arise from in-vitro culturing, such as genetic drift [41]. Additionally, studies suggest syngeneic models may not be an accurate representation of the immunological profile of human GBM. This poor immunological translatability has evinced in numerous studies whereby the efficacy of several CPIs in GL261 gliomas did not correspond to that in humans; this can likely be attributed to GL261s high mutational load compared to human GBM [43].

As the cells used are murine in origin, immunocompetent mice can be used, rendering these models particularly useful in studying immunotherapies. Furthermore, the presence of a fully functional immune system offers a more representative model of the TME. In contrast to GEMMS, syngeneic models are more easily generated in a controlled and reproducible manner, enabling consistent experimental results. Their in-vitro culturing also contributes to their cost-effectiveness and facilitates rapid tumour development, providing researchers with the flexibility to investigate a range of specific mutations, pathways, and therapeutic responses.

#### GEMMs

Genetic engineering is the process by which the genome of zygotes or embryos is modified to achieve a desired genetic outcome. Advancements in understanding the genetic alterations and mutations that drive and influence GBM have enabled the development of GEMMs. GEMMS allow researchers to replicate precise molecular and cellular characteristics of GBM in-vivo, enabling the study of tumorigenesis, and therapeutic interventions at each stage of tumour progression. GEMMs can be established via several different methods such as conditional and inducible gene targeting strategies, somatic cell gene transfer as well as, more recently, CRISPR-CAS9 technology [23]. Notably, the Cre-LoxP system is a conditional gene targeting strategy that has been used to assess the role of p53 and PTEN function in glial fibrillary acidic protein (GFAP) positive GBM and has also been hugely beneficial in trialling immunotherapies [42] A significant advantage of GEMMS is their ability to model GBM in immunocompetent mice, therefore permitting crucial in-vivo investigations of immunotherapies. Additionally, tumour progression of GEMMs (whereby tumours arise *de novo*) more closely mirrors that of human GBM as they do not involve the injection of tumour cells, therefore avoiding the associated challenges such as inflammation and disturbance of the BBB [41]. However, GEMMs require advanced breeding programmes, meaning they are expensive and time consuming. Additionally, GEMMs lack heterogeneity, a key characteristic observed in human GBM, raising concerns about their ability to truly replicate the complexity and diversity of the disease [41] (Fig. 3D).

## Organoids

Organoids are 3D self-organising structures derived from stem cells that can differentiate into tissue specific cell types, mimicking the architecture and function of their corresponding organs in-vitro [44]. Organoids can be precisely manipulated to model specific diseases, making them invaluable tools for studying disease mechanisms and drug screening. Organoid technology is a relatively recent innovation that has been widely recognised as ground-breaking, revolutionising the development of new models that more accurately represent human tissue architecture, cellular diversity, and physiological function in-vitro [45, 46].

### Generating COs

COs are generated by inducing organ-specific adult stem cells (ASCs) or pluripotent stem cells (PSCs) to form embryoid bodies (EBs) [47]. EBs are then exposed to specific growth factors and culture conditions to encourage neural differentiation. Developing neuroepithelial tissues are then transferred and embedded in a matrix (often Matrigel), before being transferred to a spinning bioreactor [46]. Over time (approximately 2–3 months), COs develop that mimic the organisation and function of the human brain [32].

PSCs, such as embryonic stem cells (ESCs) or induced pluripotent stem cells (iPSCs), are frequently used for modelling the brain [48, 49]. The development of COs from PSCs requires targeted differentiation from the guidance of growth factors, signalling molecules and cytokines. PSC derived organoids are highly structurally accurate, and mirror the mesenchymal, epithelial, and even endothelial characteristics of organs. Unfortunately, PSC derived organoids have a short proliferation period, resulting in organoids that typically resemble foetal tissue. Although this can be advantageous in certain studies, such as investigating organogenesis and embryonic development, it can be considered a drawback in the context of GBM drug screening. In contrast, ESC derived organoids can be more developed and therefore can be used as an alternative model [44]. However, the use of ESCs triggers several ethical concerns.

ASCs represent a novel approach to organoid generation [50]. Sato et al. was the first to apply 3D ASC organoid technologies to develop intestines, paving the way for the use of ASCs in other, normal and tumour, organoids [51]. ASC derived organoids require a shorter culture and more closely resemble mature tissue; however, they lack any stromal components, weakening their reliability as a cerebral model [32].

Additionally, in 2009, Ootani et al. developed the air-liquid interface (ALI) method to culture organoids; a procedure that enables the generation of organoids with epithelium and stroma [52]. This technique involves utilising Boyden chambers to culture organoids at the interface between an air and nutrient-rich medium. The organoids are positioned on a porous membrane that facilitates optimal nutrient and oxygen exchange, supporting long-term growth and maturation. The ALI method better replicates the complexities of the TME, making it particularly useful in studying diseases such as GBM [32].

### Generating GBM Organoids

**Fig. 4:**
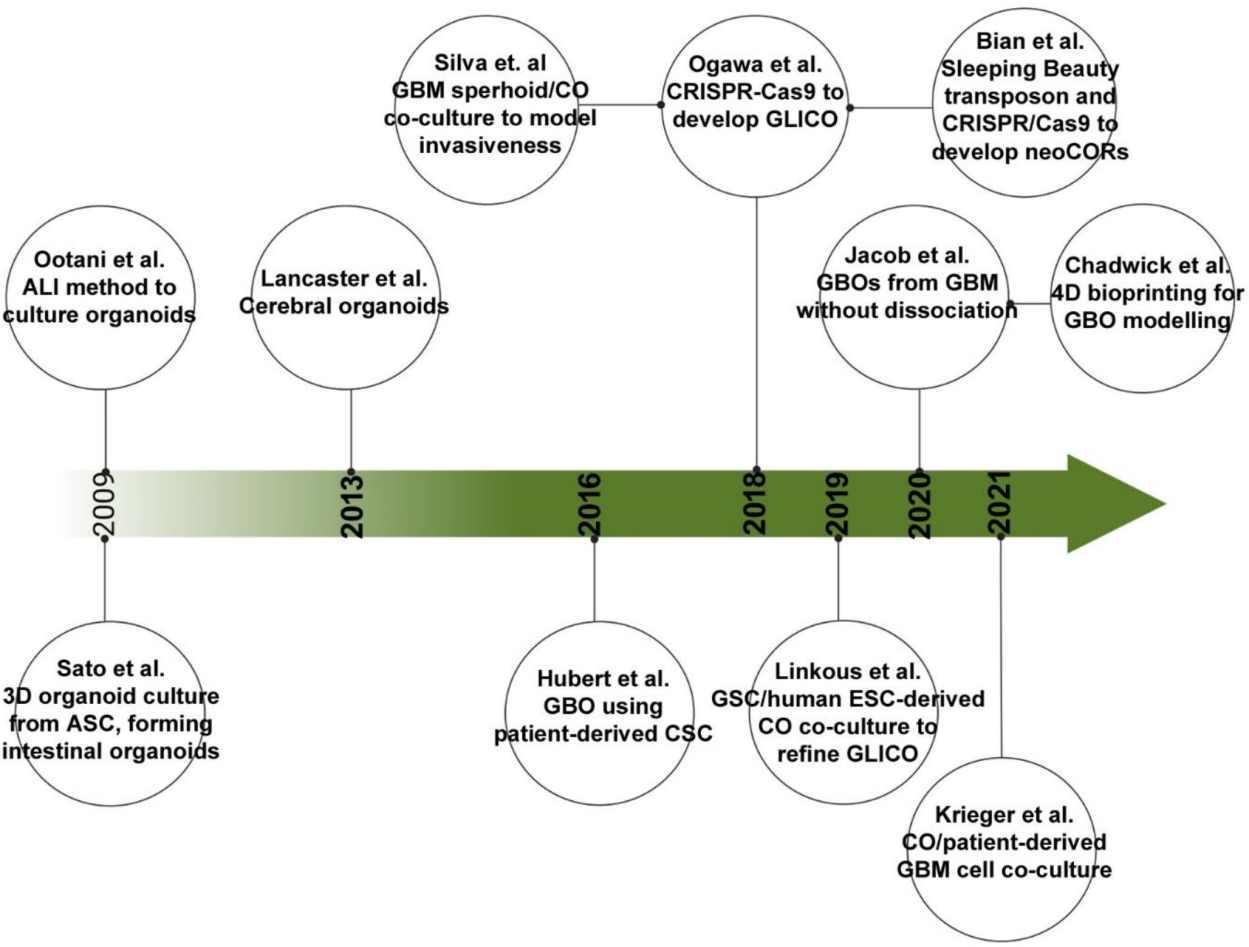
Illustrates the timeline of organoid development. A summary of key studies in the development of GBOs. Created with BioRender [37, 51, 52, 55–61]

GBM organoids (GBOs) are a novel form of 3D modelling that mimic the structure and behaviour of GBM. Importantly, GBOs can be studied in-vitro and in-vivo through transplantation into immunodeficient mice. There are four commonly used methods to develop GBO models: patient-derived approaches, genetic engineering, co-culturing and bioprinting. A timeline of organoid development to support GBM research is shown in Fig. 4.

#### Patient Derived GBOs

Culturing of patient-derived tumour biopsies or cell lines can be utilised to establish GBOs that are often referred to as ‘tumoroids’. In 2016, Hubert et al. generated GBM tumoroids directly from patient samples that were either finely minced or dissociated single cell suspensions, before being inserted into healthy brain organoids. Data from this study suggests that when orthotopically xenografted, these tumoroids maintain a population of tumour cells from the patient that can mirror GBMs infiltrative phenotype [53]. Importantly, this model replicates the structure of human GBM, with a hypoxic core defined by a low amount of SOX2 + senescent stem cells compared to a high density of highly proliferating SOX2 + senescent stem cells at the peripheries. In 2020 Jacob et al. generated GBOs directly from resected GBM samples, bypassing the need for dissociation, therefore preserving native intercellular interactions [46]. Subsequently, Jacob et al. developed an optimal chemical medium and utilised an orbital shaker to culture organoids, creating conditions that facilitate nutrient diffusion. This approach enables the development of organoids in 2–4 weeks and allows for cryopreservation, making it a practical model for GBM research and drug screening [54]. This technique is largely recognised as ground-breaking, revolutionising the approach to GBM modelling by retaining tumour heterogeneity and native microarchitecture.

#### Genetically Engineered GBOs

By genetically manipulating healthy brain organoids using CRISPR-Cas9 technology, it is possible to introduce mutations commonly associated with GBM, thereby generating a model of human GBM. Genetically engineered GBOs exhibit a transcriptomic profile similar to GBM patients as well as possessing an invasive and proliferative phenotype [45].

In 2018, two independent research groups developed the first genetically engineered GBOs using slightly different techniques. Ogawa et al. utilised CRISPR-Cas9 to insert the oncogenic mutation HRas^G12V^ and disrupt the TP53 tumour suppressor [47]. The model generated (glioma cerebral organoid (GLICO) model) was transplanted into immunodeficient mice, where it demonstrated invasive growth patterns resembling that of human GBM [55]. Around the same time, Bian et al. utilised both Sleeping Beauty transposon and CRISPR-Cas9 to introduce oncogenic and tumour suppressor gene mutations respectively, generating an in-vitro neoplastic cerebral organoid (neoCOR) [29]. Bian et al. utilised this technique to model two types of brain neoplasms: GBM and central nervous system primitive neuroectodermal (CNS-PNET) tumours. GBM neoCORs exhibited key diagnostic glial and proliferative markers, including S100β, Ki67 + and GFAP, as well as demonstrating genotypic and phenotypic profiles resembling that of GBM [56]. Unlike GLICOs, neoCORs retain a population of normal organoid cells enabling researchers to study different intercellular interactions, their role in tumour progression and microenvironment dynamics [55, 56]. GBM neoCORs were also modelled in-vivo through xenotransplantation into immunodeficient mice, and were found to maintain in-vitro characteristics, displaying glial neoplasm-like expansion [56].

#### Co-Cultures

Co-culturing approaches show promise as models that accurately mimic the impact of the GBM TME within COs. By cultivating COs alongside tumour cells or spheres, researchers can investigate the interactions between cells and the TME. Three different techniques have been established in this context. Firstly, in 2018, Silva et al. co-cultured spheroids derived from GBM cells or neural progenitors with COs to model GBM invasiveness [57]. Additionally, Linkous et al. co-cultivated GSCs and human ESC-derived COs to form a refined GLICO model in 2019 [58]. The findings indicate the GLICO model effectively replicates the phenotypic characteristics of GBM, while providing a scalable system that enables high throughput drug screening, a capability currently unattainable with in-vivo models. Based on the above findings, Krieger et al. conducted a study to investigate invasion and heterogeneity by co-cultivating human COs and patient derived GBM cells. Notably, this study identified potential interactions that could serve as novel therapeutic targets and successfully generated highly reproducible early-stage organoids, with the capabilities to offer a robust model for high throughput drug screening [59].

#### Bioprinting

Advances in bioprinting technology have enabled the development of more precise and physiologically relevant GBOs, offering enhanced platforms for drug screening. Chadwick et al. demonstrated the use of 4D bioprinting to create dynamic arrays of GBM organoids, capable of modelling interactions within the TME in a temporal manner. By incorporating patient-derived cells and ECM biomaterials, this study produced highly customisable organoids with potential for personalised drug screening. Furthermore, this approach enabled researchers to study the evolution of tumour heterogeneity and drug resistance in response to environmental changes, marking a significant advancement in modelling the complexity of GBM. While bioprinting technology in the context of GBM organoids is very recent, this approach provides a scalable and robust platform for high throughput drug screening, retaining key characteristics of the TME; therefore, bioprinting is likely to play a pivotal role in advancing GBM research [37].

### Application in Drug Screening

#### TMZ-Based Screening

Although TMZ remains the standard chemotherapeutic agent for GBM, resistance to this treatment is nearly guaranteed. Hence, there remains a need for studies investigating TMZ sensitivity and resistance mechanisms to develop strategies to overcome it. Golebiewska et al. generated GBOs by utilising surgically resected GBM tissue or patient-derived orthotopic xenografts. The tissue was mechanically minced and then seeded on agar-coated flasks, facilitating self-organisation into small organoids within two weeks [62]. The organoids produced retained the distinct genotypes of the tumours and were ideal for high throughput drug screening [63]. These organoids were treated with TMZ and various tyrosine kinase and CDK inhibitors. The results demonstrated a partial response to TMZ, with MGMT promoter methylation positive organoids more sensitive to the treatment. Importantly, this study then progressed to screen dianhydrogalactitol, which exhibited a significantly stronger response compared to TMZ, in an MGMT promoter methylation independent manner [63]. This study highlights the potential of GBOs in drug screening, demonstrating their use in advancing clinical research by enabling more precise testing.

#### Immunotherapy Screening

While GBO models often lack fully functional immune components, advancements in techniques have begun to address these limitations, enabling the investigation of some immunotherapies. For instance, Jacob et al. produced GBOs by culturing fresh tumour specimens without single cell dissociation in a culture medium. This method retains the cytoarchitecture and cellular interactions of the initial tumour, generating organoids within two weeks of culture. The organoids produced were extensively analysed to confirm that they histologically, molecularly, and genetically mirror the characteristics of GBM. Notably, Jacob et al. reported that the GBOs were able to maintain many specific elements of the TME, including hypoxia gradients, micro-vasculature, and immune cell populations. The GBOs were utilised to test a number of treatment responses in-vitro, including standard care therapy, tyrosine kinase inhibitors and CAR-T cell immunotherapy. To evaluate CAR-T cell therapy, the GBOs were co-cultured with CAR-T cells targeting EGFRvIII, whereby results exhibited selective tumour cell killing. Such findings highlight the promise of GBOs as a platform for immunotherapy screening; however, further methodological refinements are required to fully harness their potential in this field [54].

#### Molecular Targeted Therapeutic Screening

Loong et al. demonstrated the potential of GBOs in developing personalised therapies for GBM [64]. In this study, surgically resected tumour samples were utilised to generate GBOs, and targeted capture sequencing was employed to identify genetic alterations and therapeutic targets. Analysis identified mutations in the PTEN gene, indicating mTOR pathway activation. This prompted the screening of several approved GBM treatments targeting the mTOR pathway, ultimately identifying everolimus as the optimal therapy for the patient, given their tumour resistance to TMZ. This study emphasised the role of GBOs in personalised medicine, showcasing their ability to guide tailored therapy based on the unique genomic profile of the patient’s tumour. However, the high costs and resource-intensive nature of this technique limits its widespread clinical application [65].

### Challenges and Future Directions

#### Lack of Immune Components

As stated, many basic GBO models lack critical immune components of GBM. The immunosuppressive environment of GBM contributes to the development of a TME that supports tumour growth, invasion, and resistance to therapies. This property is largely driven by the immunosuppressive myeloid compartment of GBM, which includes tumour-associated macrophages and microglia that actively promote immune invasion and tumour progression [66]. The inability of GBOs to completely recapitulate this milieu restricts their ability to faithfully model the complexity of the GBM TME and its immune interactions. As a result, immunotherapy studies are restricted on GBOs due to the lack of a fully functional immune component, limiting their ability in evaluating this innovative GBM therapy modality.

Several studies have shown promising advances in overcoming this limitation, but they require further refining in order to fully represent stromal components. Firstly, the ALI method demonstrated the ability to support the development of organoids that maintain primary tumour epithelium and stromal components for an extended period [67]. However, this method requires the use of external growth factors which may limit their reproducibility. Additionally, Jacob et al. generated GBOs that maintained a heterogenous cellular composition, although the retention of immune and endothelial cells diminished over time [54, 61]. Improvements in these methods, combined with co-culturing of immune components, are likely to be pivotal in the advancement of GBOs and their future use in drug screening, particularly for immunotherapeutics.

#### Lack of Vasculature

Another key limitation of GBO models is the lack vasculature, which is a major component of the TME that sustains GSCs and promotes tumour growth and migration. Not only does this limit the ability of GBOs to model the TME, but it also restricts their size (to approximately 3–4 mm) and maturation [66].

Although vasculature has not been developed in GBOs yet, several studies have made progress in incorporating endothelial cells or vascular-like networks to enhance the model’s physiological relevance. Ham et al. was the first to develop vessel-like structures with characteristics of the BBB in organoids, achieved through early treatment with VEGF. However, this study failed to accurately represent the complete extent of GBM vasculature and exhibited a distorted open-circle morphology, likely due to the absence of blood pressure [68]. Alternative studies have utilised genetic engineering of human ESCs to induce the expression of human ETS variant 2, and co-culturing of organoids with endothelial cells [45], with both studies concluding that vascularisation of GBOs is technically feasible and would generate a more representative model by displaying key intercellular interactions [45, 69–70].

## GBM-on-a-chip

Organ-on-a-chip (OoC) technology has emerged as a ground-breaking tool for modelling human functional units by utilising primary and patient-derived cells. OoC systems mimic the structural and physiological properties of living tissues with high precision and can be adapted to model diseases. OoCs can be designed to represent a single organ (single-OoCs) or to combine multiple organs (multi-OoCs), creating more intricate networks. Single-OoCs are more frequently utilised for drug screening and investigating the underlying biological mechanisms of the disease and have led to the development of GBM-on-a-chip models.

### Generating a GBM-on-a-chip Model

**Fig. 5:**
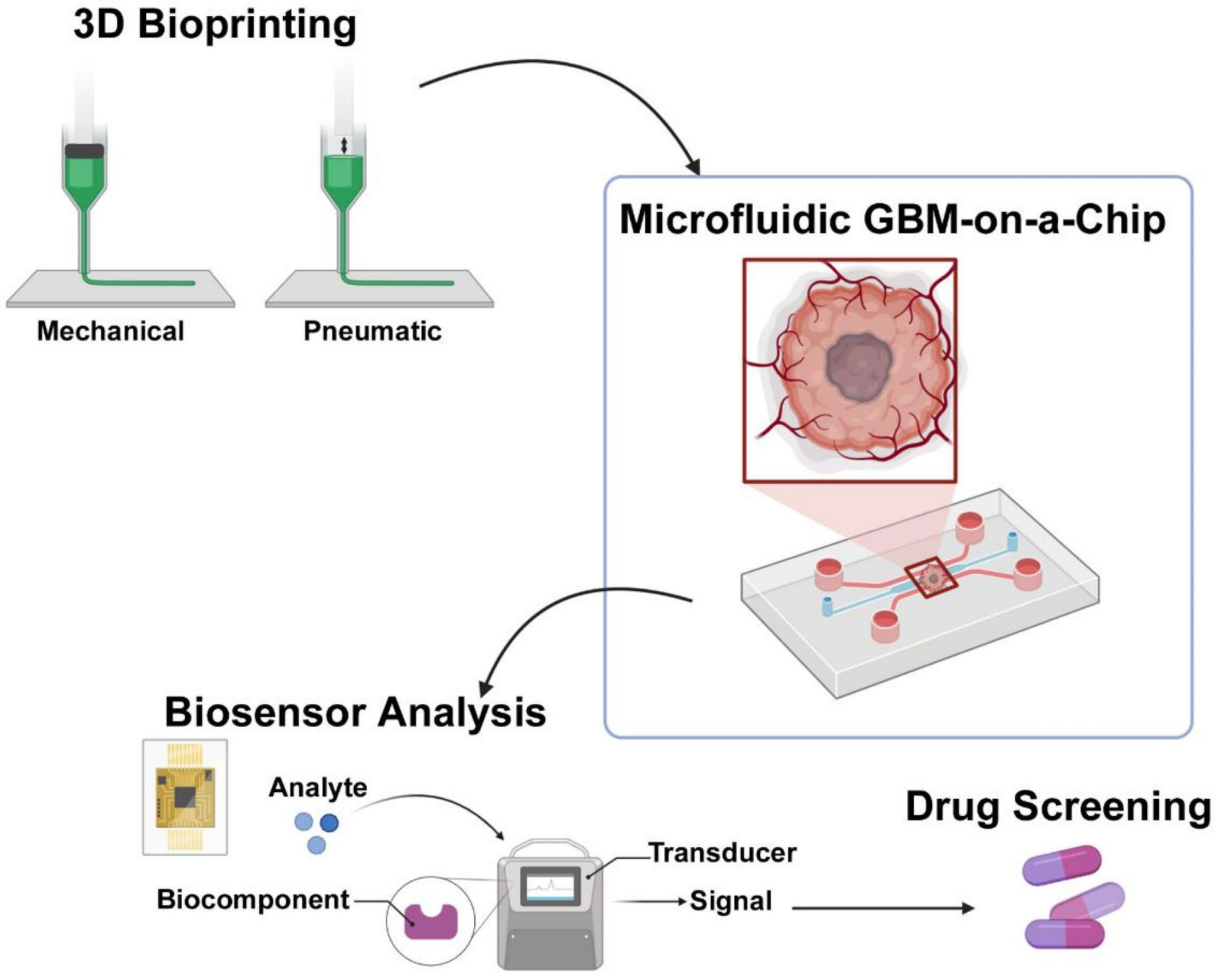
Schematic representation of a multi-platform approach for GBM modelling, incorporating 3D bioprinting, microfluidics and biosensors to enhance GBM drug screening. Created with BioRender

GBM-on-a-chip models incorporate microfluidics, bioprinting and, most recently, biosensors, to accurately replicate the TME and simulate human in-vivo physiological conditions (Fig. 5). Hence, this platform has been extensively employed to explore pharmacokinetics of both standard and novel GBM therapeutics, as well as offering promising applications in personalised medicine [71–72].

#### GBM-on-a-chip with Microfluidics

Microfluidic systems integrate micro-scale fluid channels that can mimic dynamic changes in the microenvironment, such as vasculature, immune cell infiltration and nutrient or oxygen gradients. This technology enables the co-culturing of GBM cells with other cells found in the TME, including endothelial, immune, and stromal cells, which has otherwise been restricted in traditional models [73].

Microfluidic chips are traditionally fabricated via photolithography and soft lithography using polydimethylsiloxane (PDMS) hydrogel. This involves producing a master mould using photolithography, followed by casting PDMS over the mould to form the desired microchannel structures [73]. PDMS is a transparent elastomer with favourable properties such as biocompatibility, flexibility, gas permeability and resolution [71]. Its transparency allows for real-time visualisation of cellular responses, facilitating in-depth observation of drug mechanisms [74]. Additionally, the development of valves aids the delivery of media, drugs, and signalling factors, as well as enabling researchers to manage the precise conditions and timing of fluid flow [23]. These features make PDMS-based microfluidic systems valuable for drug screening.

#### GBM-on-a-chip with Bioprinting

Bioprinting has emerged as a powerful technique in modelling GBM by generating 3D structures that replicate the complex architecture and cellular interactions of the TME. Recent advancements have enabled bioprinting to be used alongside microfluidics, generating a more intricate and reproducible GBM-on-a-chip model. The post integration technique involves bioprinting of the living parts into pre-engineered microfluidic platforms [75]. This approach allows for the precise placement of cells and tissues within the microfluidic chip and maintains distinct tumour architecture to closely mimic the in-vivo TME.

Among the various bioprinting techniques, extrusion-based bioprinting, whereby the deposition of materials through a nozzle is steady and continuous, has become a popular approach for GBM modelling, largely because of its compatibility with a wide range of bio-inks [35, 75–76]. The two primary dispensing methods for extrusion-based bioprinting are pneumatic-based and mechanical-based. Pneumatic dispensing relies on pressure changes, offering flexibility and precise control by adjusting air pressure. However, pneumatic systems can struggle with highly viscous bio-inks and often require careful calibration to avoid inconsistent flow as a result of delay in pressure changes. In contrast, mechanical dispensing utilises a piston or screw system to drive bio-inks through the nozzle, making it more suitable for high viscosity materials and providing greater levels of spatial control [76].

One of the key advantages of bioprinting technology is the ability to employ a one-step production approach, therefore enabling a range of customisable architectural designs and advanced precision. Additionally, bioprinting overcomes several challenges associated with PDMS-derived GBM-on-a-chip models, offering a less labour intensive, quicker and more precise alternative [75].

#### Biosensor-enhanced GBM-on-a-chip

The incorporation of biosensor technology in GBM-on-a-chip models marks a significant advancement in GBM research, providing real-time monitoring of biochemical and biophysical changes within the TME. Real-time data acquisition facilitates continuous, non-invasive monitoring of tumour progression, cellular metabolism, and drug responses within the TME.

Biosensors typically consist of three key components: the sensitive element, the transducer or detector module, and the signalling process [73, 77]. The sensitive element (often a biologically derived component) interacts with a specific target analyte such as a GBM associated biomarker. This interaction activates a signal that is converted into a measurable output by the transducer or detector module. The signal processor then refines and displays the signal in an interpretable form that can be analysed for insights into tumour behaviour and drug efficacy [73]. There are multiple different types of biosensors, including electrochemical, electrical, and optical, with each sensor designed to monitor specific biological factors. The effective use of biosensors in GBM-on-a-chip models requires careful consideration to ensure the properties of sensors align with specific research goals. In GBM-on-a-chip models, electrochemical biosensors are predominantly utilised due to their high sensitivity and low detection limit [73, 77]. Once the appropriate sensor is selected, the fabrication of a compatible microfluidic chip is required, along with the strategic positioning of the biosensor within the chip to optimise data acquisition and analysis. Following integration, GBM and stromal cells are seeded onto the chip to replicate the cytoarchitecture of human GBM.

Biosensor technology can be employed in two key areas of GBM research: diagnostics and drug screening. As a diagnostic tool, biosensors offer enhanced sensitivity and accuracy compared to traditional static cultures, by enabling the early detection of GBM specific biomarkers, including nucleic acids, proteins, miRNAs, circulating tumour cells, extracellular vesicles, and tumour tissues. Biosensors provide a non-invasive approach that supplements established imaging modalities for GBM diagnosis, facilitating early detection. In drug screening, biosensors support high throughput drug screening by providing instant feedback on therapeutic efficacy, reducing reliance on other time-consuming models. Furthermore, biosensor-enhanced GBM-on-a-chip technology allows for the testing of personalised therapeutic regimens, dynamically monitoring and adjusting conditions based on the unique molecular profile of the patient’s tumour [73].

### Application in Drug Screening

Multimodal systems, that incorporate microfluidics, 3D bioprinting and biosensors, have demonstrated promise in evaluating various GBM therapeutics. These advanced platforms offer a more comprehensive approach in assessing treatment responses in a physiologically representative environment. The following briefly outlines several studies that have demonstrated the potential of GBM-on-a-chip systems in drug screening.

#### TMZ-based Screening

GBM-on-a-chip models have been used to investigate the efficacy of TMZ-based therapy, as both a single and combination therapy approach. Ozturk et al. created a 3D microfluidic platform comprising of bioprinted patient derived GBM spheroids integrated within two perfused vascular channels. Using high resolution second-generation mesoscopic florescence molecular tomography imaging, the study analysed the long-term effects of TMZ treatment within this platform. The results revealed an initial decrease of tumour cells during the first three weeks of treatment; however, a subset of cells survived treatment and became invasive, consistent with the inherent TMZ resistance of GBM. Importantly, this study provided a customisable, long-term in-vitro model to investigate GBM, and emphasised the need for multi-model treatment strategies to overcome resistance and recurrence [78].

Akay et al. generated a GBM-on-a-chip model cultured from patient-derived spheroids to evaluate the combinatory therapy of TMZ and bevacizumab. The platform incorporated immunofluorescence staining of tumour specific markers to monitor treatment responses across three different patient-derived spheroids. Although the study consistently demonstrated improved efficacy with combinatory treatment, it also highlighted significant variability. This variability, a well-documented challenge, is attributed to the heterogeneity of the disease. Akay et al. proposed this chip is also capable of culturing human-derived primary cancer cells and is therefore able to offer a rapid, patient-specific platform enabling personalised therapeutics [79].

#### Immunotherapy Screening

Unlike traditional models, which often fail to recapitulate the complex interplay between the immune and vascular components with the TME, these advanced systems are able to model the immunosuppressive niche and vascular components of GBM. This capability renders GBM-on-a-chip platforms particularly advantageous for screening immunotherapies.

Cui et al. was the first to engineer a patient-derived microfluidic model that represents GBM immune and vascular components, enabling the investigation into the impact of the immunosuppressive TME, immune-vascular and cell-matrix interactions. This model was utilised to evaluate the efficacy of a novel immune CPI approach, involving the dual integrin (α_v_β_3_) and cytokine receptor (TGFß-R1) blockade. This dual target strategy suppresses GBM neovascularisation by addressing macrophage-associated immunosuppression, endothelial-macrophage interactions and altered ECM. The results from this study emphasised the role of immune and vascular modulation in GBM and demonstrated an encouraging adjuvant therapeutic option. In addition, by incorporating patient derived GBM cells, endothelial cells and tumour associated macrophages, this system offers a versatile approach for personalised therapeutic screening, as well as high throughput screening [80].

#### Nanotherapy Screening

The use of nanoparticles in facilitating drug delivery, particularly across the BBB, is a promising strategy for improving GBM treatment outcomes. GBM-on-a-chip technology facilitates the investigation of this approach, providing a valuable platform for studying the behaviour and efficacy of nanotherapeutics in a controlled, biomimetic microenvironment.

Lee et al. developed a droplet-based microfluidic platform to produce 3D tumour spheroids, capable of autonomously generating 42,000 spheroids an hour. This high throughput process involves the initial culturing of spheroids within a microfluidic device, followed by harvesting. The harvested spheroids were then utilised to evaluate the effects of photothermal therapy (PTT), mediated by a reduced graphene oxide-branched polyethyleneimine-polyethylene glycol (rGO-BPEI-PEG) nanocomposite. The results demonstrated a dose-dependent reduction in spheroid viability in response to the rGO-BPEI-PEG nanocomposite. Moreover, this study underscores the versatility of microfluidic platforms, highlighting their capability to efficiently produce tumour models for the screening of novel biomaterial-based therapies [81].

#### Drug Compound Screening

Fan et al. developed a novel GBM-on-a-chip model, constructed from photo-polymerizable poly(ethylene) glycol diacrylate (PEDGA) hydrogel, with the specific aim of enhancing drug screening. The GBM cell line U87 was cultured to generate a reproducible model that successfully recapitulated key features of the disease, with potential to transform high throughput GBM drug screening. This model was then utilised to screen the dual treatment of pitavastatin and irinotecan, two frequently used anti-cancer drugs. The results showed a decrease in cell survival by at least 50% in all cell channels and suggest that this model provides an adjustable and stable platform for high throughput screening, with experimental results generated as early as four days post treatment. It is also important to recognise that the developed structure of the 3D model accurately replicates the hypoxic core formed in early human tumour development. Furthermore, an additional advantage of this approach is its ability to overcome the lengthy fabrication process of PDMS-based microfluidics, enabling the generation of a model in under two hours [82].

### Challenges and Future Directions

#### PDMS-Related Challenges

While PDMS-derived microfluidics has revolutionised OoC technology, it does have several limitations. Ongoing research is focused on addressing these limitations and identifying the most effective methods and materials to accurately model GBM-on-a-chip.

Firstly, the low manufacturing efficiency of PDMS-based microfluidic devices restricts their scalability and widespread implementation in research [71]. Secondly, although the hydrophobic and porous nature of PDMS is beneficial for preventing fluid leaking and enabling the creation of oxygen and nutrient gradients, it also presents several complications. One significant issue is the absorption of small, hydrophobic molecules, including lipids and some drugs, into the PDMS material [23, 83]. This can lead to inconsistencies in the concentrations of these substances within the microfluidic system, affecting the accuracy and reproducibility of experimental results. Additionally, it could alter drug efficacy or interfere with cellular responses, complicating drug screening and the study of therapeutic mechanisms in PDMS-derived GBM-on-a-chip-models. To overcome this, the culture media should be replaced frequently. Moreover, continuous addition of nutrients is required to prevent the rapid depletion of nutrients and premature build-up of metabolites. Additional PDMS-related challenges include gas and fluid evaporation under poor humidity conditions, toxicity if not properly cured, and cell attachment problems [23].

#### Bioprinting-Related Challenges

A major limitation of bioprinting is the demand for multi-material manufacturing capabilities, as accurately recreating the complexity of the TME typically requires the simultaneous use of different bio-inks. Moreover, the development of suitable bio-inks remains a significant challenge; bio-inks must be compatible with the cellular components of the TME and maintain their biological properties overtime. Further research is required to refine bio-ink formulations and bioprinting techniques to ensure they can model the intricate dynamic nature of GBM in a reproducible and scalable manner [75].

#### Detection and Sampling Challenges

Although the small size of cell culture chambers minimises reagent use and enables high throughput screening, it restricts the number of cells and results in challenges with sampling and detection of secreted products. Additionally, the continuous perfusion of culture medium, essential for cell viability, further dilutes these secretions, amplifying the issue. To address this, careful design of channels and perfusion rates is necessary, with possible solutions including elongating culture chambers, reducing flow rates or increasing the number of parallel microchannels [84].

#### Cell Source and Heterogeneity

The choice of cell sources is key in generating realistic GBM-on-a-chip platforms. The incorporation of immortalised cell lines into chip models is common, due to their availability and ease of culture; but they lack patient-specificity, limiting their application. Primary patient-derived cells, sourced from biopsies, better preserve the unique biological characteristics of the tumour, enabling the creation of personalised models. This personalised approach facilitates the screening of therapeutic regimens that are more predictive of patient responses. Nevertheless, inconsistent cultures, time constraints and difficulty obtaining these samples restricts their use in these models. GSCs are another valuable cell source, as they can be maintained for relatively long culture periods and can differentiate into many cell types, closely recapitulating the heterogeneity observed in GBM. However, isolating and maintaining GSCs in culture is technically challenging, hence their integration into GBM-on-a-chip system remains limited. Consequently, these models may not be able to fully capture the heterogeneity of GBM [85].

Alternative strategies to address these limitations include the incorporation of spheroids (or more recently, organoids) cultured outside the chip system. This synergistic approach can address the limitations of both models, whilst combining their unique advantages. This is a recent progression and faces many challenges, particularly with the development of new microfluidic devices that can accommodate organoids. However, the successful incorporation and development of these models could prove revolutionary for GBM research [86].

## Conclusion

GBM is a highly aggressive and heterogenous cancer, and despite extensive global multidisciplinary research efforts, there is limited effective treatment and patient prognosis remains poor. This highlights the need for more representative preclinical models to enable high throughput screening of standard and novel therapeutics. A summary of traditional and next-generation models for GBM is shown in Table 2.

Traditional preclinical models have progressed to more accurately model GBM but still face significant limitations in fully replicating the complexity of the disease. In-vitro models, though easily manipulated and controlled, often fail to recreate the TME and cytoarchitecture. In contrast, in-vivo models capture GBM in a living organism and therefore preserve more key cellular interactions, as well as maintaining the tumour and surrounding structure. However, in-vivo models often lack the immune complexity and tumour heterogeneity of human GBM. As such, these limitations, along with the lack of an effective treatment, emphasise the need for more representative models to enhance clinical translation and improve therapeutic outcomes.

Next-generation models, such as GBOs and GBM-on-a-chip platforms, offer promising solutions to these challenges. GBOs provide a better recapitulation of GBM heterogeneity, including the maintenance of distinct populations of cells through co-culturing. Alternatively, microfluidics is able to represent the TME in a controlled and adjustable manner, accurately simulating fluid flow, nutrient gradients and the mechanical forces that influence tumour behaviour. By integrating organoid cultures in microfluidic devices, it is possible to create a more accurate, synergistic model that consolidates the advantages of each model, allowing for a more comprehensive investigation.

Importantly, the ability to utilise patient-derived cells in both GBOs and GBM-on-a-chip systems enables the creation of more personalised models that reflect the individual GBM TME. By maintaining patient specific heterogeneity, these models facilitate the investigation of how different patients may respond to various treatments, offering a more tailored approach to therapeutic optimisation. This personalisation enhances the potential for more effective individualised therapeutic strategies, ultimately advancing the field of precision medicine and improving the clinical relevance of GBM preclinical research.

**Table 2:** Summary of traditional and next-generation GBM models

Model	Technological integration	Specific Applications in Drug Screening	Throughput and Scalability	Cost and Time Efficiency	Model Complexity	Advantages	Disadvantages
Established cell lines			High	High	Low	Easy to handle Reproducible	Poor representation of in-vivo conditions, Lack of TME, Genetic and phenotypic drift
Primary patient cell lines		Personalised drug screening	Medium	Moderate; challenging to establish	Low to medium	Maintains some patient characteristics	High variability, Short viability
Spheroids			Medium	Moderate	Medium	Preserves 3D cytoarchitecture	Lacks full TME
Scaffolds			Medium	Low	Medium	Customisable, Provide support and structure	Lack full TME and heterogeneity
Pre-mortem brain samples			Very low	Low; limited availability and high cost / time investment	Medium	Maintains native TME	Lack of standardisation, Lack of heterogeneity
Post-mortem brain samples		Studying end-stage disease	Very low	Low; limited availability and high cost / time investment	Medium	Maintain native TME, Provide insight to end-stage disease	Limited availability, High costs, Technically challenging
Organotypic brain slice cultures			Low	Low; limited availability and high cost / time investment	High	Maintain native TME, Manipulatable	Can lack full TME, Scalability issues
Cell line xenografts			Medium	Moderate	Medium	Easy to establish and manipulate	Immunosuppressed mice, Lacks human TME
Patient-derived xenografts		Personalised drug screening	Low	Low	Medium	Maintains TME and some heterogeneity	Immunosuppressed mice, Lacks human TME
Syngeneic mouse models		Immunotherapeutics	Medium	Moderate	Medium	Immunocompetent mice can be used	Limited translational application as murine specific tumours
Genetically engineered mouse models	CRISPR-Cas9, Transgenics	Tumorigenesis, Immunotherapeutics	Low to medium	Low	High	Mimics genetic alterations and tumorigenesis, Immunocompetent mice can be used	Expensive, Time consuming, Lacks human TME
Organoids	Bioprinting		Medium	Moderate	High	Mimics 3D architecture, heterogeneity, and intercellular interactions	Limited vascularisation and stromal components, Lack of standardisation
Glioblastoma-on-a-chip	Microfluidics, Bioprinting, Biosensors	Testing drug responses under dynamic conditions	Medium	Moderate	Very High	Recreates dynamic TME conditions, Real time monitoring	Technically challenging, Expensive, Lack of standardisation

A current major limitation of GBOs and GBM-on-a-chip models is the lack of standardised protocols, as these technologies are very recent techniques and are constantly evolving to improve their accuracy. Additionally, GBOs are highly sensitive to even minor changes, resulting in a high level of inter-organoid variability, making it difficult to evaluate the validity of these studies. A variety of techniques have been introduced in an attempt to improve reproducibility within GBO studies including engineered ECM-like materials over Matrigel scaffolds, microfluidics, and miniaturised spinning bioreactors which allow better availability of differentiation signals to the culture [45, 87]. Similarly, the inconsistency between GBM-on-a-chip models makes it difficult to compare data across studies. The incorporation of machine learning techniques, such as biosensors, can enhance the quality and reproducibility of these models [75, 85]. However, further optimisation of these techniques is required to enable their widespread use and reliance as a drug screening model. Despite these challenges, it is clear GBO and GBM-on-a-chip models will play a pivotal role in the advancement of GBM therapies, potentially improving overall patient outcomes.

## Key References

- Yesudhas D, Dharshini SAP, Taguchi YH, Gromiha MM. Tumor Heterogeneity and Molecular Characteristics of Glioblastoma Revealed by Single-Cell RNA-Seq Data Analysis. Genes (Basel). 2022;13(3). 10.3390/genes13030428.
  - (Outstanding Importance) This study elegantly exemplifies the degree of GBM genetic heterogeneity and thereby the challenge to model such diversity using preclinical models.
- Slika H, Karimov Z, Alimonti P, Abou-Mrad T, De Fazio E, Alomari S, et al. Preclinical Models and Technologies in Glioblastoma Research: Evolution, Current State, and Future Avenues. Int J Mol Sci. 2023;24(22). 10.3390/ijms242216316.
  - (Of Importance) This review highlights current and next-generation preclinical models for GBM, with an emphasis on humanized and immunocompetent murine models.
- Pasupuleti V, Vora L, Prasad R, Nandakumar DN, Khatri DK. Glioblastoma preclinical models: Strengths and weaknesses. Biochim Biophys Acta Rev Cancer. 2024;1879(1):189,059. 10.1016/j.bbcan.2023.189059.
  - (Of Importance) This review highlights current and next-generation preclinical models for GBM, with an emphasis on humanized and immunocompetent murine models.
  - This review highlights GBM preclinical modes and recommends that an idealised model should establish a functional relationship between driver oncogenes and biological responses.
- Watanabe F, Hollingsworth EW, Bartley JM, Wisehart L, Desai R, Hartlaub AM, et al. Patient-derived organoids recapitulate glioma-intrinsic immune program and progenitor populations of glioblastoma. PNAS Nexus. 2024;3(2):pgae051. 10.1093/pnasnexus/pgae051.
  - (Outstanding Importance) This study characterised genetic, metabolic and immune profiles of GBM patient-derived organoids, revealing an intrinsic immune-like landscape utilised by glioma stem cells.
- Alves AH, Nucci MP, Mamani JB, Valle NME, Ribeiro EF, Rego GNA, et al. The Advances in Glioblastoma On-a-Chip for Therapy Approaches. Cancers (Basel). 2022;14(4). 10.3390/cancers14040869.
  - (Outstanding Importance) This review highlights current and next-generation preclinical models for GBM, with an emphasis on humanized and immunocompetent murine models.
  - This systematic review summarises recent advances in GBM organ-on-a-chip and microfluidic devices to assess preclinical therapeutic assessment.

## Data Availability

No datasets were generated or analysed during the current study.
